# Prostate tuberculosis complicated by huge prostatic abscess: A rare case report from Nepal

**DOI:** 10.1016/j.ijscr.2020.10.045

**Published:** 2020-11-02

**Authors:** Suman Baral, Raj Kumar Chhetri, Milan Gyawali, Neeraj Thapa, Ranjit Mahato, Rupesh Sharma, Prahar Dahal

**Affiliations:** a Department of Surgery, Lumbini Medical College and Teaching Hospital Ltd Tansen-11, Pravas, Palpa, Nepal; b Department of Radiology, Lumbini Medical College and Teaching Hospital Ltd Tansen-11, Pravas, Palpa, Nepal; c Department of Pathology, Lumbini Medical College and Teaching Hospital Ltd Tansen-11, Pravas, Palpa, Nepal

**Keywords:** Prostatic abscess, Prostatic tuberculosis, Trans-urethral loop drainage

## Abstract

**Introduction:**

Prostatic tuberculosis is one of the rarest findings in clinical practice and associated prostatic abscess is even scarce, described in literatures. We present a rare case of prostatic tuberculosis complicated by huge prostatic abscess.

**Presentation of a case:**

A 68-year-old male with no any comorbidity presented with history of increased frequency of micturition along with poor flow, urgency and nocturia for 17 days. He was under medical treatment for benign enlargement of prostate for 2 years. Per rectal examination revealed a boggy cystic swelling anteriorly with enlarged prostate with mild tenderness. Ultrasonography abdomen and pelvis showed massive enlargement of prostate with central avascular necrotic area with moving internal echoes. Contrast enhanced computed tomography (CECT) showed 230 g of prostate with central liquefaction of approximately 101 mm^3^. Transurethral loop drainage along with resection of prostate was done. Histopathology revealed granulomatous prostatitis highly suggestive of prostatic tuberculosis. Prostatic abscess culture was negative. Patient is currently under category 1 anti-tubercular therapy.

**Discussion:**

Prostatic tuberculosis is a rare clinical finding which is commonly seen in patients with disseminated tuberculosis with immunocompromised status. Prostatic abscess in setting of granulomatous tuberculosis of prostate is even rarer. Transrectal ultrasonography is the investigation of choice for diagnosis of abscess if available. Treatment includes drainage of abscess preferably transurethral, and antitubercular therapy.

**Conclusion:**

Trans-urethral loop drainage is a safe treatment modality for patients presenting with prostatic abscess followed by anti-tubercular therapy if histopathology findings are suggestive of granulomatous lesions.

## Highlights

• Prostatic tuberculosis is one of the rarest clinical entities.
• Huge prostatic abscess might be one of the complications of granulomatous prostatitis.
• Most cases follow the disseminated tuberculosis.
• Transurethral loop drainage can be a promising method of treatment.
• Category 1 anti-tubercular therapy should be initiated.

## 1 Introduction

Genitourinary tuberculosis (GUTB) accounts for 30–40% of extrapulmonary tuberculosis (EPTB), second to lymph nodes while 15–20% of pulmonary tuberculosis lands with GUTB in developing countries [1]. Prostate tuberculosis (PTB) which is quiet uncommon disease is usually diagnosed on histopathology while undergoing transurethral resection. Renal, vesiculo-seminal and epididymal tuberculosis are more common followed by prostate involvement which occurs through hematogenous routes, descending infection from the urinary organs and direct intracanalicular extension from a neighboring tuberculous focus. Tuberculous abscesses of the prostate are infrequent, and generally found only in immunocompromised patients associated with HIV and AIDS infection [2]. We present an unusual case of huge prostatic abscess with benign enlargement of prostate in otherwise healthy male managed with transurethral loop resection of prostate and drainage of abscess with no evidence of other primaries or dissemination. This work has been reported in accordance to the Surgical Case Report (SCARE) guidelines [3].

## 2 Presentation of a case

**Fig. 1:**
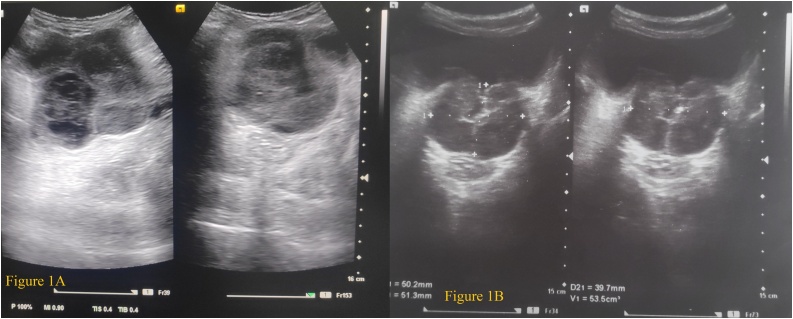
(A) Huge prostate with central avascular necrotic area that demonstrates moving internal echoes within suggestive of prostatic abscess. (B) Decreasing prostatic volume to approximately 53.5 mm^3^.

A 68-year-old male presented to outpatient department of surgery with history of increased frequency of micturition for 17 days associated with urinary dribbling, poor flow which didn’t improve while straining, urgency and nocturia. The patient was a known case of Benign Enlargement of Prostate (BEP) and has been under medication with tamsulosin 0.4 mg HS (taken at bed time) and finasteride 5 mg OD (once daily) for last 2 years. There was no history of hematuria, fever, pain abdomen and difficulty breathing. The patient had no history of urethral instrumentation, surgical intervention, past history of tuberculosis among family members or any chronic illness in the past. On clinical examination, vitals were stable. Chest and abdominal examination findings were normal. Per rectal examination revealed a boggy swelling anteriorly, which was cystic in consistency along with massively enlarged prostate that was slightly tender was appreciated. Laboratory examinations were normal with increased level of prostate specific antigen (PSA) level of 5.64 ng/mL. Routine urine examination showed pus cells of 10–12/high power field (HPF). Foleys catheterization was done and patient was admitted. Transrectal ultrasonography being unavailable, transabdominal ultrasonography (USG) revealed massive enlargement of prostate with central avascular necrotic area showing moving internal echoes (Fig. 1A).

**Fig. 2:**
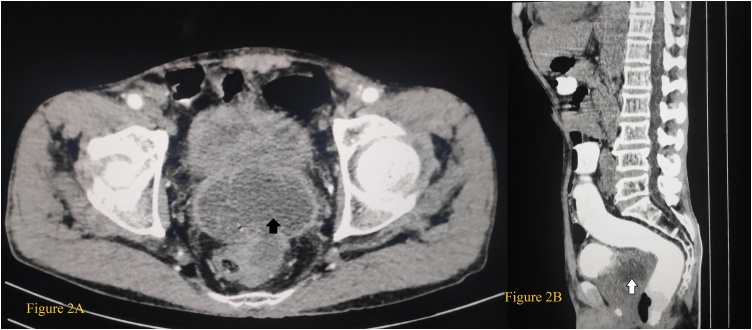
(A) The axial section of the abdomen and pelvis with a black arrow head that demonstrates massively enlarged prostate measuring about 230 g with central liquefaction of approximately 101 mm^3^. (B) The coronal section with white arrow head showing the prostatic abscess.

**Fig. 3:**
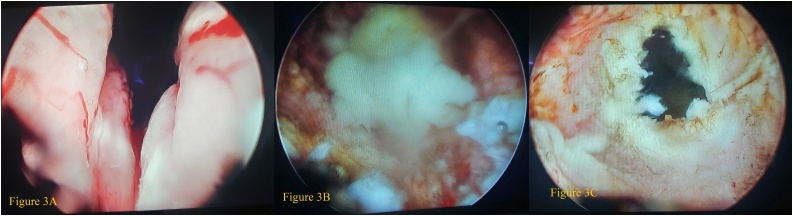
(A) The bilobar enlargement of the prostate. (B) The efflux of pus from the abscess cavity. (C) The deroofing of the abscess cavity.

**Fig. 4:**
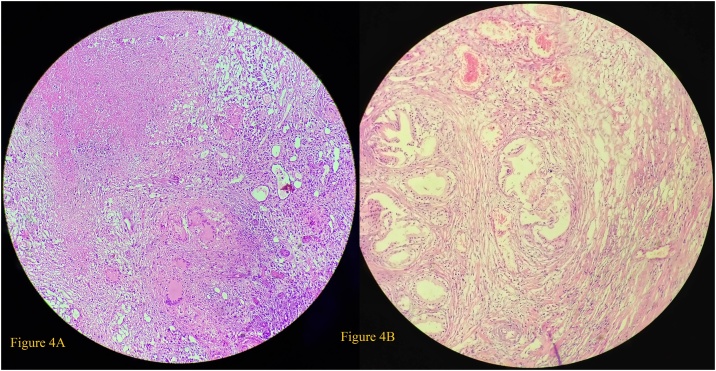
(A) and (B) The H&E stained sections which show discrete as well as confluent well-formed epithelioid granulomas with caseation and Langhan’s type of giant cells. Dense multifocal necrotic areas are evident along with increased proliferation of stromal and glandular components that show double layered epithelium- inner columnar and outer cuboidal to flattened epithelium suggestive of benign prostatic hyperplasia.

Urine culture showed growth of enterococcus species and Vancomycin with Cefoperazone-sulbactum was started in the view of prostatic abscess. Contrast Enhanced Computed Tomography (CECT) abdomen showed massively enlarged prostate measuring about 230 g with central liquefaction of approximately 101 mm^3^ along with significant adjacent inflammatory changes (Fig. 2A and B). Six days after admission when urine culture was sterile, transurethral drainage of the abscess was planned by the surgical team and prepared for surgery accordingly. Intraoperatively, bi-lobar enlargement of prostate along with high bladder neck was appreciated (Fig. 3A). The prostatic abscess could not easily be delineated whilst resection of the enlarged prostate was carried out. Meanwhile, intraoperative per-rectal manipulation of prostate visualized the pus point and puncture of the prostate was done into it along with gentle transrectal massage of the prostate which provided the counter force to expel the pus from the cavity (Fig. 3B and C). About 60 g of prostatic tissue was removed. Patient was catheterized again, put on intravenous antibiotics. Pus culture sensitivity was sterile. Sixth day post operatively, the patient was discharged for home-based care after removal of catheter on oral antibiotics with linezolid which was sensitive preoperatively for next 7 days. Histopathology examination of prostatic specimen showed well-formed epithelioid granulomas with caseation and Langhan’s type of giant cell suggestive of necrotizing granulomatous prostatitis highly suggestive of prostatic tuberculosis (Fig. 4A and B).

**Fig. 5:**
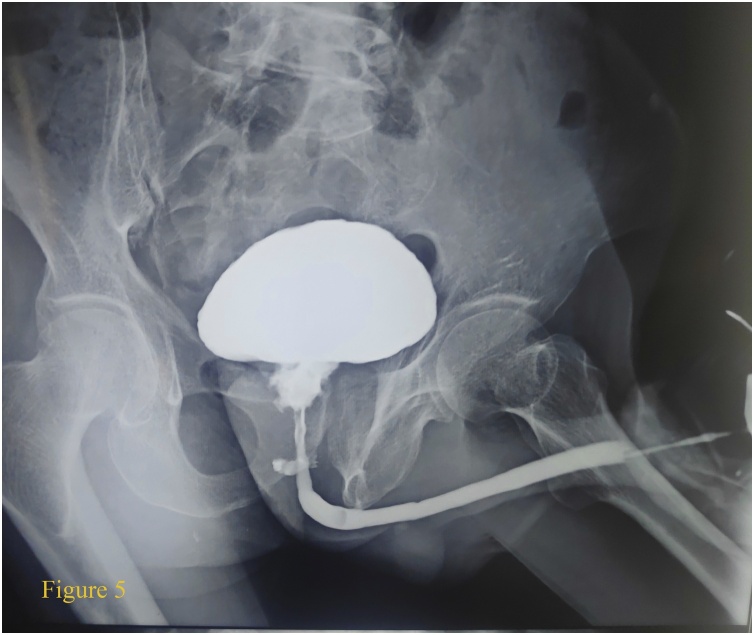
The Micturition Cystourethrogram (MCUG) with normal opacification of urinary bladder with irregular wall and ill-defined prostatic urethra with loss of normal outline and no evidence of peritoneal spillage.

Follow up examination after 2 weeks showed the urinary symptoms of the patient improving with no evidence of rectal-urethral fistula as evidenced by micturition cystourethrogram (MCUG) in view of unavailability of Magnetic Resonance Imaging (MRI). (Fig. 5) Repeat transabdominal USG showed 53.5 g of prostate (Fig. 1B). Mantoux tuberculin skin test was normal along with serum adenosine deaminase (ADA). Sputum acid fast bacilli (AFB) was negative. Patient was then started on category 1 antitubercular therapy with Isoniazid, Rifampicin, Pyrazinamide and Ethambutol (HRZE) in the view of extrapulmonary tuberculosis. No evidence of other primaries was found concluding tuberculosis to be prostatic primary. The second follow up after 2 months of initiation therapy of ATT was devoid of side effects and patient compliance was good.

## 3 Discussion

TB affects almost all the genital tracts that include seminal vesicles, vas deferens, epididymis, prostate, testicles, cooper glands and penis. Prostatic involvement is quite rare which is less than 5% of all genitourinary organs and many of the treating surgeons will have difficulty regarding management of the entity as most of the incidental findings of PTB is met upon while undergoing transurethral resection [4]. In an autopsy conducted by Spoorer et al. among 728 disseminated cases, PTB was delineated in 100 subjects which included prostatic involvement in the form of granulomatous prostatitis and prostatic abscess [5].

Predisposing factors responsible for PTB include immunosuppressives, steroid use and immunocompromised states [6]. Upon presentation, PTB usually manifest as granulomatous prostatitis whilst abscess formation is exceedingly rare as mentioned in the literatures. Till date, only 21 cases have been articulated mostly from India, Spain and 5 cases from United States [7]. Among those few reported cases of abscess, most of them were associated with HIV/AIDS [8].

Majority of patients may present with features of frequency, urgency, nocturia along with poor flow, however 25% of the cases may not reproduce any symptoms [9]. Our patient had the above described symptoms for about 3 weeks duration along with oral medications for the enlarged prostate. PTB may be diagnosed upon the histopathology of the prostatic specimen after transurethral resection as clinical evidences might not be sufficient to make preoperative judgement. However, per rectal examination might provide a clue for an experienced clinician as cystic mass might be elucidated for complicated PTB with abscess. Granulomatous changes if associated with prostatic abscess might be picked upon in transabdominal or transrectal USG (TRUS) with sensitivity being higher of the latter that involves direct contact with the prostate and resolution by intervening surfaces is not limited. Also, abscesses most commonly appear as hypoechoic (anechoic to isoechoic) areas, often of varying sizes containing thick liquid with internal septations. TRUS is contraindicated in patients with anal fistulas and severe hemorrhoids, and can be highly painful for some patients with prostatic abscess. CECT abdomen and pelvis seems to be the diagnostic modality of choice for emphysematous abscess, as it allows for clear visualization of gas and fluid accumulation in the prostate gland [10]. Pus culture sensitivity shows most commonly gram-negative organisms mainly *Escherichia coli* and *Staphylococcus aureus* being commoner for abscess developed via hematogenous routes. Other reported organisms include *Klebsiella pneumoniae*, *Enterococcus* species, *Pseudomonas aeruginosa*, *Bukholderia pseudomalle* and *Brucella melitensis*. Atypical organisms have been reported in immunocompromised and HIV/AIDS patients that includes *Mycobacterium* as well as fungal lesions including *Coccidioides immitis*, *Candida* species and *Histoplasma capsulatum* [10]. MRI which shows better inherent tissue contrast resolution than CT and abscesses show iso- to hypointense signal with peripheral contrast enhancement on T1-weighted images, and heterogeneous hyperintense signal on T2-weighted images. Still, overall sensitivity for the diagnosis still favors for TRUS and this has been regarded the diagnostic of choice [10].

Small sized abscesses may be amenable for out-patient based treatment with antibiotics like ciprofloxacin in the view of no appropriate treatment guidelines being released. But, the concerns of developing resistance in view of abscesses caused by atypical organisms like mycobacterium has created to follow the judicial selection of antibiotics along with less threshold for undergoing for surgical drainage in case conservative approach fails. However in-patient management is warranted if there are chances of developing sepsis. Parenteral regimens might include a third-generation cephalosporin, aztreonam or the combination of an aminoglycoside with ampicillin. Antibiotics alone should only be attempted in stable patients with abscesses less than 1 cm with serial imaging for the resolution of the abscess [11]. TRUS guided aspiration of the abscess might be the first line of therapy as most of the treating surgeons are comfortable with it and success rates are very high though some chances of incomplete drainage or recurrence do exist in cases with multiloculated and thick pus which mandates transurethral deroofing of the cyst. Deroofing approach seems to be promising in cases with BEP along with recurrent or residual abscesses. This approach has shown the recurrence rate of as low as 7% in patients who has previously undergone deroofing [12]. Loop drainage along with use of holmium laser for deroofing has been mentioned in literatures. Lee at al. mentioned his paper regarding use of Holmium laser for deroofing in 8 patients with no single recurrence, however this modality may not be feasible in low resource settings like ours where the cost factor plays a significant role in patient management [13]. Open perineal drainage can be the other treatment modality in cases with extra prostatic involvement which may not be advocated in era of minimal invasive surgery which is associated with significant risk of impotence along with septicemia related poor wound healing and potential for superinfection [10]. Cases with tubercular abscess, once drained should be started on antitubercular therapy in the view of extrapulmonary tuberculosis. Disseminated tuberculosis should be kept in mind and primaries should be sought for. We believe, this case report with PTB along with huge abscess is one of the rarest variants as there were no evidences of distant primaries. Successful management of such huge abscess transurethrally with no evidence of urethrorectal fistula upon follow up justifies the treatment we provided. We hope this case scenario will be the valuable addition to the scarce amount of literatures related to such presentations of prostatic tuberculosis along with huge abscess especially in low income countries where prevalence of tuberculosis is high. If treating surgeons are dealing with such scenarios and sterile pyuria is evident, prostatic tuberculosis ought to be one of the differentials.

## 4 Conclusion

Prostatic abscess with sterile pyuria should arise index of suspicion of prostatic tuberculosis. Transurethral deroofing can be easily adopted with caution as a safe measure for the management of larger abscesses.

## Declaration of Competing Interest

The authors report no declarations of interest.

## Sources of funding

This case report did not receive any specific grant from funding agencies in the public, commercial, or not-for-profit sectors.

## Ethical approval

Ethical approval was not mandatory for publication of case reports as per the institutional policy.

## Consent

“Written informed consent was obtained from the patient for publication of this case report and accompanying images. A copy of the written consent is available for review by the Editor-in-Chief of this journal on request”.

## Author contribution

Design and Idea: Suman Baral, Raj Kumar Chhetri, Rupesh Sharma.

Drafting: Suman Baral, Ranjit Mahato.

Final Revision: Suman Baral, Raj Kumar Chhetri, Milan Gyawali, Neeraj Thapa, Prahar Dahal, Rupesh Sharma, Ranjit Mahato.

## Registration of research studies

NA.

## Guarantor

Suman Baral.

## Provenance and peer review

Not commissioned, externally peer-reviewed.
